# A blood-based liquid biopsy analyzing soluble immune checkpoints and cytokines identifies distinct neuroendocrine tumors

**DOI:** 10.1186/s13046-025-03337-3

**Published:** 2025-03-05

**Authors:** Pablo Mata-Martínez, Lucía Celada, Francisco J. Cueto, Gonzalo Sáenz de Santa María, Jaime Fernández, Verónica Terrón-Arcos, Nuria Valdés, Vanesa García Moreira, María Isabel Enguita del Toro, Eduardo López-Collazo, María-Dolores Chiara, Carlos del Fresno

**Affiliations:** 1 The Innate Immune Response Group, La Paz University Hospital Research Institute (IdiPAZ), Paseo de La Castellana 261, Madrid, 28046 Spain; 2 Immunomodulation Laboratory, La Paz University Hospital Research Institute (IdiPAZ), Madrid, Spain; 3 Health Research Institute of the Principado de Asturias (ISPA), Av. de Roma S/N, Oviedo, 33011 Spain; 4 Institute of Oncology of the Principado de Asturias, University of Oviedo, Oviedo, Spain; 5 Current Address: Fundación Idonial, Gijón, Spain; 6 Tumor Immunology Laboratory, La Paz University Hospital Research Institute (IdiPAZ), Madrid, Spain; 7 Endocrinology and Nutrition Department, Hospital Universitario Cruces, Biobizkaia, UPV/EHU, CIBERDEM, CIBERER, Endo-ERN, Barakaldo, Bizkaia Spain; 8 Clinical Analysis Service, San Agustín University Hospital, Avilés, Spain; 9 Clinical Analysis Service, Central University Hospital of Asturias, Oviedo, Spain; 10 CIBERES (Network of Biomedical Research in Respiratory Diseases), Madrid, Spain; 11 Universidad UNIE, Madrid, Spain

**Keywords:** Soluble immune checkpoint, Neuroendocrine neoplasm, Immunological factor, Liquid biopsy

## Abstract

**Background:**

Neuroendocrine neoplasms (NENs) comprise a group of rare tumors originating from neuroendocrine cells, which are present in both endocrine glands and scattered throughout the body. Due to their scarcity and absence of specific markers, diagnosing NENs remains a complex challenge. Therefore, new biomarkers are required, ideally, in easy-to-obtain blood samples.

**Methods:**

A panel of blood soluble immune checkpoints (sPD-L1, sPD-L2, sPD-1, sCD25, sTIM3, sLAG3, Galectin-9, sCD27, sB7.2 and sSIGLEC5) and cytokines (IL4, IL6, IP10 and MCP1) was quantified in a cohort of 139 NENs, including 29 pituitary NENs, 46 pheochromocytomas and paragangliomas, and 67 gastroenteropancreatic and pulmonary (GEPP) NENs, as well as in 64 healthy volunteers (HVs). The potential of these circulating immunological parameters to distinguish NENs from HVs, differentiate among various NENs subtypes, and predict their prognosis was evaluated using mathematical regression models. These immunological factors-based models generated scores that were evaluated by Receiver Operating Characteristic (ROC) and Area Under the Curve (AUC) analyses. Correlations between these scores and clinical data were performed. From these analyses, a minimal signature emerged, comprising the five shared immunological factors across the models: sCD25, sPD-L2, sTIM3, sLAG3, and Galectin-9. This refined signature was evaluated, validated, and checked for specificity against non-neuroendocrine tumors, demonstrating its potential as a clinically relevant tool for identifying distinct NENs.

**Results:**

Most of the immunological factors analyzed showed specific expression patterns among different NENs. Scores based on signatures of these factors identified NENs with high efficiency, showing AUCs ranging between 0.948 and 0.993 depending on the comparison, and accuracies between 92.52% and 95.74%. These scores illustrated biological features of NENs including the similarity between pheochromocytomas and paragangliomas, the divergence between gastrointestinal and pulmonary NENs, and correlated with clinical features. Furthermore, the models demonstrated strong performance in distinguishing metastatic and *exitus* GEPP NENs, achieving sensitivities and specificities ranging from 80.95% to 88.89%. Additionally, an easy-to-implement minimal signature successfully identified all analyzed NENs with AUC values exceeding 0.900, and accuracies between 84.11% and 93.12%, which was internally validated by a discovery and validation randomization strategy. These findings highlight the effectiveness of the models and minimal signature in accurately diagnosing and differentiating NENs.

**Conclusions:**

The analysis of soluble immunological factors in blood presents a promising liquid biopsy approach for identifying NENs, delivering critical insights for both prognosis and diagnosis. This study serves as a proof-of-concept for an innovative clinical tool that holds the potential to transform the management of these rare malignancies, providing a non-invasive and effective method for early detection and disease monitoring.

**Supplementary Information:**

The online version contains supplementary material available at 10.1186/s13046-025-03337-3.

## Background

Neuroendocrine neoplasms (NENs) encompass a diverse group of rare malignancies accounting for around 0.5% of all newly diagnosed tumors [1]. Although their incidence is low, estimated at 1–5 cases per 100,000 inhabitants annually, the number of reported diagnoses has been increasing over the past decade, probably due to longer lifespan, increased awareness and the widespread use of imaging diagnostic procedures [2, 3]. The term neuroendocrine surges from widely dispersed cells all around the body identified by both neuronal-like and endocrine properties. They are called “neuro” because, similarly as neurons, neuroendocrine tumor cells show dense core granules and immunoreactivity to neuronal markers such as chromogranin A (CgA) or neuron-specific enolase (NsE). The term “endocrine” comes from their capacity to produce amine hormones [4, 5]. In terms of location, neuroendocrine cells can be found in endocrine glands, such as the pituitary or the adrenal gland, as well as scattered along the digestive and respiratory tracts, constituting the diffuse endocrine system [5]. The two predominant types are gastroenteropancreatic (GEP-NENs) and those affecting the respiratory tract, grouped as gastroenteropancreatic and pulmonary NENs (GEPPs) [3]. Nevertheless, NENs can also develop in less common sites such as the adrenal glands and extra-adrenal paraganglia, referred to as pheochromocytomas (PCCs) and paragangliomas (PGLs), collectively known as PPGLs, or the pituitary gland, named as pituitary NENs (Pit-NEN) [1]. Regardless of their organ of origin, common features of all NENs are the secretion of peptide hormones and/or biogenic amines, and the expression of neuroendocrine markers [6]. Despite these common features, there is significant diversity in their histology, molecular profiles, and clinical manifestations. With the aim to standardize this complexity, in 2022 the World Health Organization (WHO) proposed a classification of endocrine and neuroendocrine tumors based on their differentiation, proliferative grading and location [7].

In terms of clinical behavior, NENs present a wide spectrum of symptoms, complicating both diagnosis and treatment [8]. Hormone-secreting NENs can often be identified by symptoms resulting from hypersecretion of specific hormones by neuroendocrine cells [9]. However, their clinical presentation can be nonspecific, potentially leading to diagnostic errors. Conversely, patients with non-secreting NENs present heterogeneous clinical profiles, either lacking early-stage symptoms or presenting with nonspecific symptoms, further complicating an accurate diagnosis [6]. Therefore, developing diagnostic biomarkers is crucial for early-stage tumor detection.

While tumor tissue markers of neuroendocrine differentiation, such as CgA and synaptophysin, are available in clinical practice, they require invasive biopsy and immunohistopathological evaluation [10]. Alternatively, blood-based analyses offer a non-invasive and easily accessible approach. Classic examples of circulating biomarkers include CgA or NsE, and hormones associated with hormone-secreting syndromes (e.g., insulin for insulinomas, gastrin for gastrinomas, and metanephrines in PPGLs) [8, 11–13]. However, general biomarkers cannot distinguish between different NENs, while hormone markers are only useful in some hormone-secreting NENs, limiting their overall diagnostic utility. Furthermore, CgA is secreted in numerous non-NEN-related pathologic conditions and demonstrates limited sensitivity and specificity [12, 13]. Therefore, there is an unmet need for circulating blood biomarkers for early NEN diagnosis.

Recent studies indicate that the assessment of circulating immune proteins holds promise for enhancing early diagnostic accuracy, prognosis and to predict the response to immunotherapy across various cancer types [14–17]. Despite these advancements, the exploration of blood-circulating biomarkers in NENs remains quite limited. Only few studies have evaluated immune-related factors in blood such as the neutrophil-to-lymphocyte ratio or the systemic immune-inflammation index (considering neutrophil, lymphocyte and platelet counts) as diagnostic and prognostic markers in GEP-NENs [18–21]. Of note, results obtained from diverse experimental approaches in NEN tissue sections indicate differential immunological behaviors, with potential implications in prognosis and new immunotherapy-based treatments [22–26]. Nevertheless, the diagnostic and prognostic value of circulating immune checkpoint molecules and cytokines from easy-to-obtain blood samples in NENs remains largely unexplored.

This study aimed to investigate whether patients suffering from NENs exhibit altered levels of circulating immune checkpoint proteins, potentially serving as diagnostic or prognostic biomarkers. To this end, we analyzed multiple soluble immune checkpoints and cytokines in a large cohort of NEN patients, including PPGLs, GEPPs and Pit-NENs. Our findings demonstrate that scores generated from combinations of these circulating immune markers can effectively discriminate patients with NENs from healthy individuals, as well as distinguish between different NEN subtypes. These scores also allow an efficient identification of GEPPs progressing to metastasis and *exitus*. Eventually, we propose a minimal signature based on five soluble immune checkpoints (sCD25, sPD-L2, sTIM3, sLAG3, Galectin-9) as a potential liquid biopsy approach to identify any of the analyzed NENs. These findings could provide a novel and robust diagnostic tool for these rare malignancies, potentially aiding in the identification of patients who may benefit from immunotherapy.

## Methods

### Recruitment of healthy volunteers and patients

This study was conducted following the ethical guidelines of the 1975 Declaration of Helsinki and was approved by the local ethics committee of La Paz University Hospital (PI-5270). Clinical data available were used to cluster the patients (*n* = 139) in different groups according to the NEN they were suffering from: Pheochromocytomas and paraganglyomas, (PPGLs, *n* = 43), Gastroenteropancreatic and pulmonary NENs (GEPPs, *n* = 67) and Pituitary NENs (Pit-NENs, *n* = 29).

Samples from these patients were recruited during the period 2011 – 2023 at the Central University Hospital of Asturias and University Hospital of Cabueñes (total samples = 46; PPGLs = 18, GEPPs = 22, Pit-NENs = 6), and at the Principado de Asturias BioBank (PT20/0161) (total samples = 93; PPGLs = 25, GEPPs = 45, Pit-NENs = 23). This biobank is integrated into the Spanish National Biobanks and Biomodels Network. Noteworthy, samples were pathologically classified based on the 2022 WHO Classification of Endocrine and Neuroendocrine Tumors [7]. Healthy volunteers (HVs, *n* = 64) were recruited from the Blood Donation Unit at La Paz University Hospital.

A second cohort of patients was studied including 36 HVs also recruited from the Blood Donation Unit, 55 non-small cell lung carcinoma (NSCLC) patients, and 27 luminal breast cancer patients recruited, respectively, from the Thoracic Surgery and the Radiodiagnostic departments of La Paz University Hospital.

All samples were processed following standard operating procedures with the appropriate approval of the Ethical and Scientific Committees. Demographic and clinical data of HVs and patients are included in Table 1 and Supplementary Table 1.

**Table 1:** Demographic and clinical data of participants

Variable	n	%
Number of samples	203	100
Patients	139	68.47
Healthy volunteers	64	31.53
Age in years, median (min–max)		
Patients	58.50 (28–81)	-
Healthy volunteers	57.00 (30–67)	-
Sex		
Patients	139	68.47
Male	82	58.99
Female	57	41.01
Healthy volunteers	64	31.53
Male	41	64.06
Female	23	35.94
Pathology		
Neuroendocrine neoplasms (NENs)	139	
Pit-NENs	29	20.86
Male	21	72.42
Female	8	27.58
PPGLs	43	30.93
Male	19	44.18
Female	24	55.82
Pheochromocytomas	25	58.14
Paragangliomas	18	41.86
GEPP NENs	67	48.21
Male	42	62.68
Female	25	37.32
Metastatic	31	46.27
No Metastatic	24	35.82
No data	12	17.91
Gastrointestinal NENs	22	32.84
Metastatic	12	54.55
No metastatic	4	18.18
No data	6	27.27
Pancreatic NENs	36	53.73
Metastatic	13	36.11
No metastatic	17	47.22
No data	6	16.67
Pulmonary NENs	9	13.43
Metastatic	6	66.67
No metastatic	3	33.33
Clinical features		
Pit-NENs		
Differentiation status		
Well-differentiated	29	100
PPGLs		
Differentiation status		
Well-differentiated	2	4.65
Poorly differentiated	22	51.16
No data	19	44.19
GEPPs		
Grade		
Grade I	26	38.81
Grade II	20	29.85
Grade III	11	16.42
No data	10	14.92
Stage		
Stage I	22	32.84
Stage II	4	5.97
Stage III	1	1.49
Stage IV	30	44.78
No data	10	14.92

*Abbreviations*: *NENs* Neuroendocrine neoplasms, *Pit-NENs* Pituitary neuroendocrine neoplasms, *PPGLs* Pheochromocytomas and paragangliomas, *GEPP* Gastroenteropancreatic and pulmonary

### Blood processing

Blood from HVs were collected in K_3_EDTA coated tubes (REF:13,060 Vacutest™ Kima) and gel & clot activator tubes (REF:10,313 Vacutest™ Kima). Soluble blood fractions were obtained after the centrifugation for 10 min at 10.000 rpm, aliquoted and conserved at -80 °C until their analysis.

### LEGENDplex analysis

Levels of circulating soluble immune checkpoints and cytokines were analyzed using a cytometry bead-based assay from BioLegend (immune checkpoint panel 1 Ref: 740,867, and essential immune response panel 13-plex Ref: 740,930, respectively). These kits included a panel of a pre-established set of soluble immune checkpoints (sCD25, s4-1BB, sCD27, sCD86 (B7.2), TGF-β1, sCTLA-4, sPD-L1, sPD-L2, sPD-1, sTIM3, sLAG3, Galectin-9) and cytokines (IL4, IL2, CXCL10 (IP10), IL1β, TNF, CCL2 (MCP1), IL17A, IL6, IL10, IFN-γ, IL12p70, CXCL8 (IL8), TGF-β1). The quantifications were performed following the manufacturer instructions. Briefly, samples were twofold diluted in assay buffer and incubated for 2 h at room temperature with the coated beads. After that time, the beads were centrifuged, washed and incubated with the detection antibodies (conjugated to biotin) for one hour at room temperature. Finally, the beads were incubated with streptavidin–phycoerythrin for 20 min at room temperature, centrifuged and washed before flow cytometry.

### sSIGLEC5 and chromogranin A ELISA

Human SIGLEC5 ELISA (Enzyme-linked immunoassay) kit (Sigma Ref: RAB0433) and human CgA Duoset ELISA kit (R&D Systems Ref: DY9098) were used. Following the instructions provided in the kit, after overnight coating plates with capture antibody at 4º (for CgA ELISA), samples were incubated at room temperature for 2.5 h, followed by 4 washes with 300 µl of wash solution. Detection antibody was incubated for 1 h at room temperature and washed 4 times with 300 µl of wash solution. Finally, 100 µl of TMB One-Step substrate reagent were added to each well and, after 30 min the reaction was stopped by adding 50 µl of stop solution to each well. The absorbance was measured at 450 nm. Each sample was 250-fold diluted for sSIGLEC5 and 20-fold for CgA determinations.

### Statistical analysis

In all panels, dots represent individuals. Data of single soluble parameters are depicted as violins plots; lines inside the violins delimitate quartiles. D’Agostino and Pearson Normality test was performed to all the variables included in the study. Considering that data, one-way ANOVA followed by Fisher’s test or Kruskal–Wallis’ test followed by uncorrected Dunn’s test were performed for more than two groups comparison, and T-test or Mann–Whitney test for two groups comparison. Spearman linear regressions were performed to establish correlations.

The backward Wald method was used to generate binary logistic regression models using SPSS version 29 (IBM) software. The Wald automatic stepwise selection method starts from the complete set of independent variables to be studied and, iteratively removes and, if necessary, reintroduces them at each step based on their statistical significance until only a set of explanatory ones remains [27]. Based on this logistic regression model, classifying scores were generated as previously described [16, 28].

Each retained variable in the model is assigned a coefficient (B factor) indicating its contribution to class differentiation. Of note, this B factor represents a “weighting parameter”, having higher, lower, positive, or negative values depending on the model, aiming to maximize the classifying capacity of the final model. Considering the concentrations of the soluble parameters included in every model, and the B factor provided by the algorithm associated with each of them, scores were generated following the formula: Score = ([parameter A] x B factor A) + ([parameter B] x B factor B) + … + ([parameter X] x B factor X). It means that a composite score is computed by summing the products of each retained parameter value and its corresponding B factor. These scores were used to generate the Receiver Operating Characteristic (ROC) curves, determining the Area Under the Curve (AUC) and optimal cut-off values as the Youden index. Whenever possible, optimal regression models were selected applying the criterium of AUC > 0.90, using the minimum number of parameters to build the model. In those cases of AUC < 0.90, the model generating the highest AUC was chosen. The AUC, sensitivity, and specificity are shown as well as their 95% confidence intervals.

Randomization in discovery and validation cohorts was conducted considering the proportional representation of the various cohorts comprising the total sample size. Specifically, for the 64 HVs, 45 samples were allocated to the discovery cohort and 19 to the validation cohort. Similarly, within the NENs group, which includes 139 samples, 91 were assigned to the discovery cohort and 48 to the validation cohort. Furthermore, the NENs group encompasses six subgroups (Pit-NENs, pheochromocytomas, paragangliomas, gastroenteric, pulmonary, and pancreatic GEPPs). The allocation of samples for each subgroup to either the discovery or validation cohorts was performed proportionally. Lastly, given that the samples were obtained from two sources (Central University Hospital of Asturias and University Hospital of Cabueñes, and the Principado de Asturias BioBank), the distribution was structured to reflect the proportions contributed by each source, ensuring an equitable and representative allocation across all cohorts.

Accuracy was determined as the number of (true positive + true negative) / number of individuals included in each analysis.

All along the figures, *p*-values (*p*) are denoted as *p < 0.05, ** p < 0.01, *** p < 0.001, **** p < 0.0001. Only variables showing statistically significant differences are depicted for clarity reasons. Statistical analyses were conducted using Prism 8.0 (GraphPad).

## Results

### Evaluation of circulating cytokines and soluble immune checkpoints in patients suffering from different neuroendocrine neoplasms

**Fig. 1:**
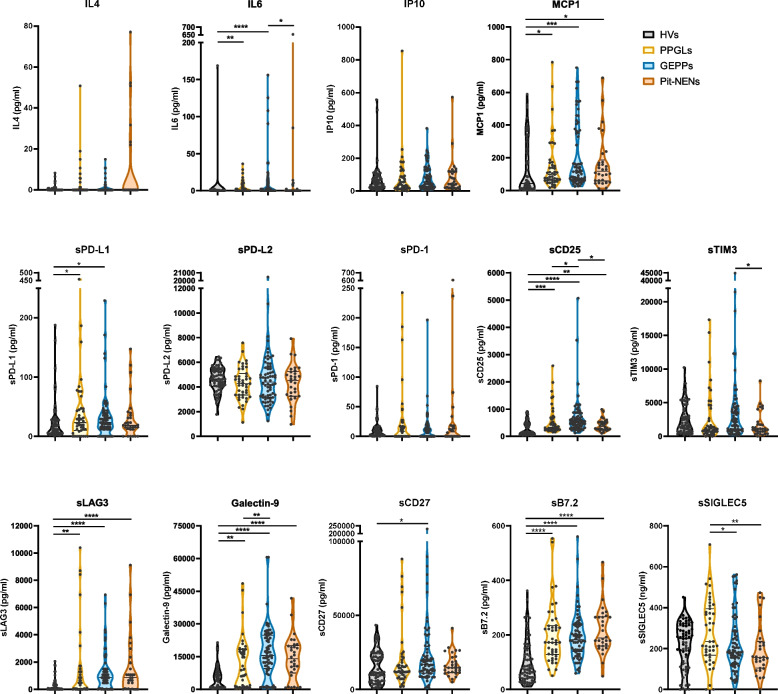
Circulating levels of cytokines and soluble immune checkpoints in patients suffering from different neuroendocrine neoplasms. The concentration of the cytokines IL4, IL6, IP10, MCP1 and the soluble immune checkpoints sPD-L1, sPD-L2, sPD-1, sCD25, sTIM3, sLAG3, Galectin-9, sCD27, sB7.2 and sSIGLEC5 was analyzed in blood soluble fractions from healthy volunteers (HVs) and patients diagnosed with pheochromocytomas and paragangliomas (PPGLs), gastroenteropancreatic and pulmonary (GEPPs) neuroendocrine neoplasms (NENs) and pituitary NENs (Pit-NENs). Data are shown as violin plots showing quartiles. Comparisons between the four groups of individuals were performed by Kruskal–Wallis’ test. *p*-values are represented as *p < 0.05, ** p < 0.01, *** p < 0.001, **** p < 0.0001

To investigate whether the presence of NENs influences the expression of circulating immunological factors, we evaluated the circulating levels of a panel of cytokines (IL4, IL2, CXCL10 (IP10), IL1β, TNF, CCL2 (MCP1), IL17A, IL6, IL10, IFN-γ, IL12p70, CXCL8 (IL-8), TGF-β1) and soluble immune checkpoints (sCD25, s4-1BB, sCD27, sCD86 (B7.2), TGF-β1, sCTLA-4, sPD-L1, sPD-L2, sPD-1, sTIM3, sLAG3, Galectin-9 and sSIGLEC5) in samples from patients suffering from PPGLs, GEPPs and Pit-NENs. Given that the patient cohort for this study was sourced from two distinct sub-cohorts—Central University Hospital of Asturias and University Hospital of Cabueñes, and the Principado de Asturias BioBank—, an initial quality assessment was conducted. This involved examining whether the levels of soluble immune checkpoints and cytokines were comparable across sub-cohorts for each specific NEN. Subsequently, we considered those immunological factors that consistently showed detectable levels of expression (over the detection limit). These technical analyses (data not shown) defined IL4, IL6, IP10 and MCP1, along with sPD-L1, sPD-L2, sPD-1, sCD25, sTIM3, sLAG3, Galectin-9, sCD27, sB7.2 and sSIGLEC5 as robust immunological factors to be included in the study (Fig. 1).

Among the cytokines, MCP1 levels were significantly elevated in PPGLs, GEPPs and Pit-NENs compared to HVs (Fig. 1). This consistent increase across all NENs was also observed in the soluble immune checkpoints sCD25, sLAG3, Galectin-9 and sB7.2. Notably, specific expression profiles emerged for IL6, sPD-L1, sTIM3, sCD27 and sSIGLEC5 across the different neoplasms (Fig. 1). Specifically, elevated IL6 and sPD-L1 distinguished PPGLs from HVs, while GEPPs were characterized by increased levels of IL6, sPD-L1, and sCD27. Among the NENs, PPGLs showed higher expression of sSIGLEC5. Increased sCD25 expression differentiated GEPPs, which also exhibited higher Galectin-9 levels than PPGLs and increased IL6 and sTIM3 compared to Pit-NENs (Fig. 1).

This first analysis indicates the existence of specific immunological patterns that could differentiate HVs from NENs, as well as differentiate between different types of NENs.

### Differential expression pattern of circulating cytokines and soluble immune checkpoints between grouped neuroendocrine neoplasms

Having observed specific expression patterns concerning grouped NENs, we moved to study potential differences between neoplasms classified as PPGLs and GEPPs.

**Fig. 2:**
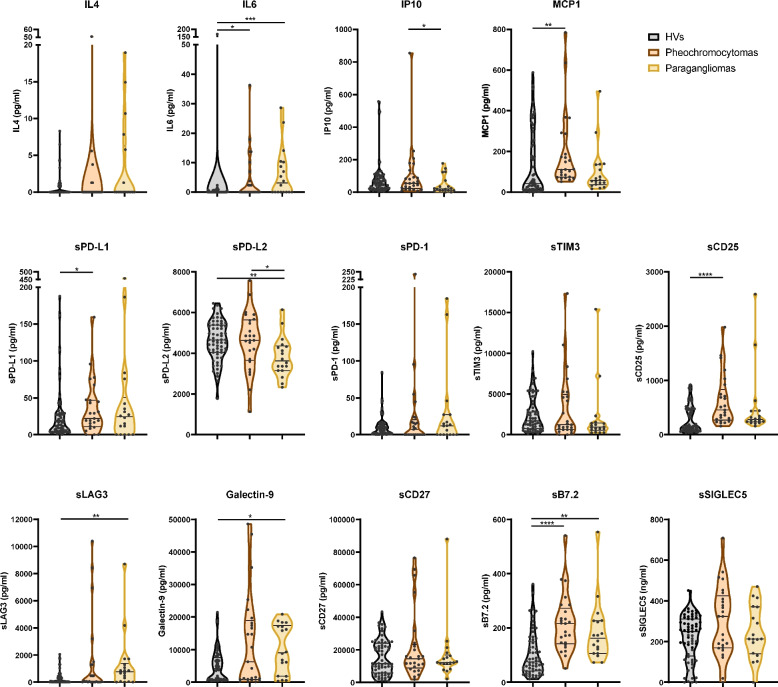
Peripheral blood levels of cytokines and soluble immune checkpoints in patients suffering from pheochromocytomas or paragangliomas. The concentration of the cytokines IL4, IL6, IP10, MCP1 and the soluble immune checkpoints sPD-L1, sPD-L2, sPD-1, sCD25, sTIM3, sLAG3, Galectin-9, sCD27, sB7.2 and sSIGLEC5 was analyzed in blood soluble fractions from healthy volunteers (HVs) and patients diagnosed with pheochromocytomas or paragangliomas. Data are shown as violin plots showing quartiles. Comparisons between the four groups of individuals were performed by Kruskal–Wallis’ test or one-way ANOVA (for sPD-L2). *p*-values are represented as *p < 0.05, ** p < 0.01, *** p < 0.001, **** p < 0.0001

As indicated before, pheochromocytomas and paragangliomas are clustered as PPGLs. Beyond the common increased IL6 and sB7.2 expression levels displayed by both tumor entities compared to HVs (Fig. 2), pheochromocytomas showed heightened MCP1, sPD-L1 and sCD25 levels, while paragangliomas exhibited reduced sPD-L2 expression with increased levels of sLAG3 and Galectin-9. Interestingly, only IP10 and sPD-L2 were different between pheochromocytomas and paragangliomas, with reduced expression of both factors in paragangliomas (Fig. 2).

**Fig. 3:**
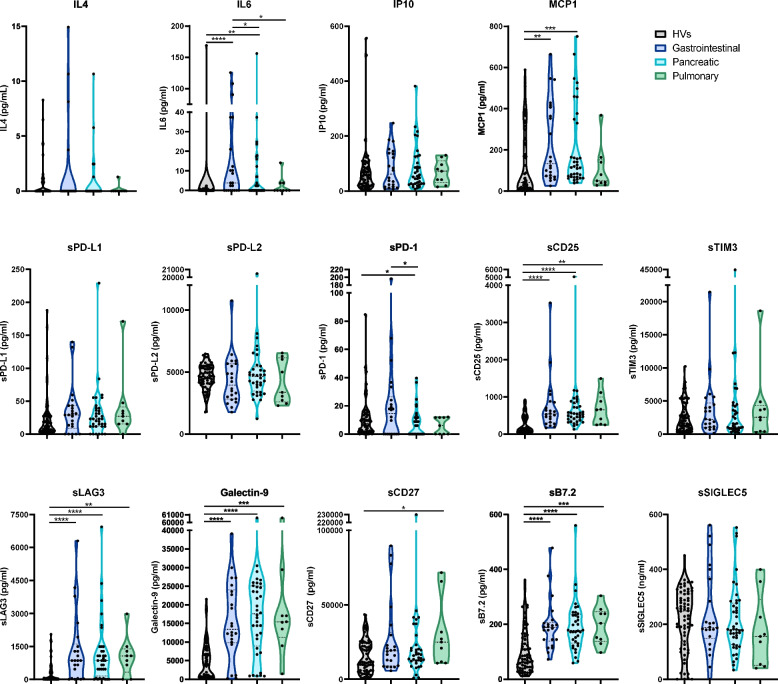
Circulating levels of cytokines and soluble immune checkpoints in patients suffering from gastroenteropancreatic and pulmonary neuroendocrine neoplasms. The concentration of the cytokines IL4, IL6, IP10, MCP1 and the soluble immune checkpoints sPD-L1, sPD-L2, sPD-1, sCD25, sTIM3, sLAG3, Galectin-9, sCD27, sB7.2 and sSIGLEC5 was analyzed in blood soluble fractions from healthy volunteers (HVs) and patients diagnosed with gastrointestinal, pancreatic or pulmonary neuroendocrine neoplasms. Data are shown as violin plots showing quartiles. Comparisons between the four groups of individuals were performed by Kruskal–Wallis’ test. *p*-values are represented as *p < 0.05, ** p < 0.01, *** p < 0.001, **** p < 0.0001

GEPPs include gastrointestinal and pancreatic NENs, as well as those arising at the pulmonary tract. Compared to HVs, all three neoplasms showed an increased sCD25, sLAG3, Galectin-9 and sB7.2 expression, while gastrointestinal and pancreatic NENs shared higher levels of IL6 and MCP-1 (Fig. 3). Individually, pancreatic NENs showed reduced sPD-1 levels, and sCD27 expression was increased in pulmonary NENs (Fig. 3).

These findings suggest a degree of specificity in the circulating immunological profiles within the defined groups, with each neoplasm exhibiting characteristic patterns. Pheochromocytomas and paragangliomas differ only in IP10 and sPD-L2 levels. Additionally, gastrointestinal and pancreatic NENs form a distinct cluster, showing differences from pulmonary neoplasms, with sPD-1 levels being the sole distinguishing factor between the gastrointestinal and pancreatic subtypes.

### Combination of circulating immunological factors efficiently identifies neuroendocrine neoplasms

Based on the observations above, we wanted to evaluate the predictive potential of soluble immune checkpoints and cytokines in circulation to identify NENs. With this aim, we performed a binary logistic regression model including the fourteen immunological variables. The generated scores were assessed by Receiver Operating Characteristic (ROC) curves, determining the Area Under the Curve (AUC), and the optimal cut-off score, estimated by the Youden index [16, 28].

**Fig. 4:**
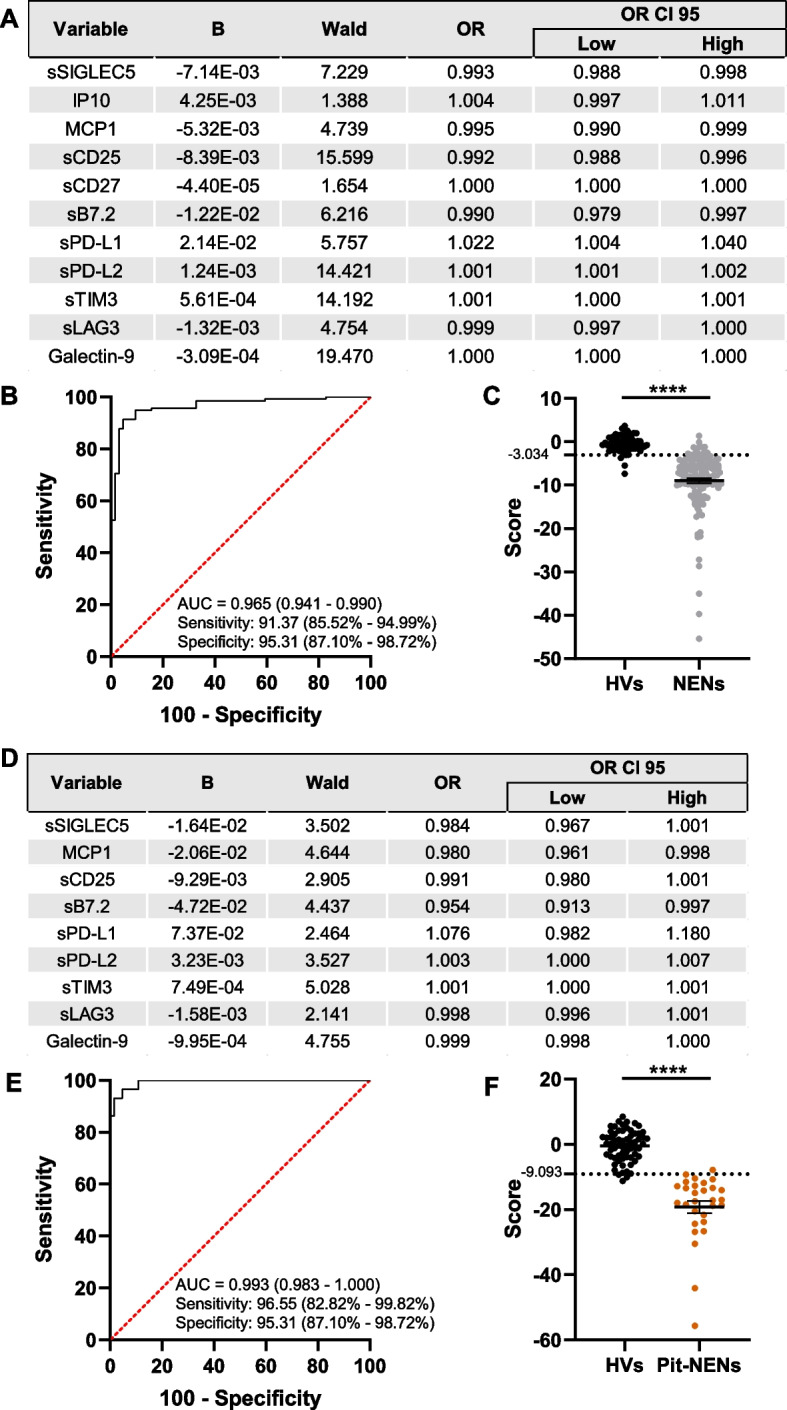
Regression models of circulating immunological factors identify neuroendocrine neoplasms. Wald backward stepwise regressions were performed, including IL4, IL6, IP10, MCP1, sPD-L1, sPD-L2, sPD-1, sCD25, sTIM3, sLAG3, Galectin-9, sCD27, sB7.2 and sSIGLEC5 as variables. **A** Optimal model differentiating neuroendocrine neoplasms (NENs) from healthy volunteers (HVs). **B** ROC curve analysis for identification of NENs. **C** Distribution of HVs and patients diagnosed with NENs according to the score generated as the optimal Youden index from the ROC curve in **B**. **D** Optimal model differentiating pituitary NENs (Pit-NENs) from healthy volunteers (HVs). **E** ROC curve analysis for identification of Pit-NENs. **F** Distribution of HVs and patients diagnosed with Pit-NENs according to the score generated as the optimal Youden index from the ROC curve in **E**. Area under the curves (AUC) are shown in **B** and **E**, as well as sensitivity and specificity. The 95% confidence intervals are shown in brackets. **** *p* < 0.0001, Mann–Whitney test

We started comparing HVs (*n* = 64) *versus* the complete cohort of NENs (*n* = 139). The regression model generated a score including eleven variables (Fig. 4A). The ROC analysis showed that this immunological signature effectively identifies NENs with an excellent performance (AUC = 0.965; 95% CI, 0.941 – 0.990) (Fig. 4B); the optimal cut-off exhibited 91.37% sensitivity (95% CI, 85.52% – 94.99%) and 95.31% specificity (95% CI, 87.10% – 98.72%) (Fig. 4B, C).

Considering this powerful identification capacity, we aimed to develop a simplified predictive signature using a reduced number of parameters, trying to enhance the technical feasibility of detection. With this approach, we developed a regression model that included only those statistically different immunological factors between HVs and NENs (see Fig. 1). These factors comprised IL6, MCP1, sPD-L1, sCD25, sLAG3, Galectin-9, sCD27 and sB7.2.

The best model included six soluble immune checkpoints (Supplementary Fig. 1A) with an AUC of 0.920 (95% CI, 0.883 – 0.958), identifying NENs with an 82.73% sensitivity (95% CI, 75.59% – 88.11%) and 89.06% specificity (95% CI, 79.10% – 94.60%) (Supplementary Fig. 1B, C). Therefore, even a compact signature performs efficiently to identify patients suffering from NENs.

Next, we sought to assess this procedure to determine whether Pit-NENs could be identified as a cohort of uniform neoplasms. The regression model included nine immunological soluble factors to differentiate HVs from Pit-NENs (Fig. 4D). This model rendered a high performance, with an AUC of 0.993 (95% CI, 0.983 – 1.000) (Fig. 4E), 96.55% sensitivity (95% CI, 82.82% – 99.82%) and 95.31% specificity (95% CI, 87.10% – 98.72%) (Fig. 4E, F). Of note, a regression model that only included the five statistically different variables between HVs and Pit-NENs (MCP1, sCD25, sLAG3, Galectin-9 and sB7.2) (Supplementary Fig. 1D) also identified Pit-NENs proficiently, with an AUC of 0.950 (95% CI, 0.910 – 0.990) that delivered a 96.55% sensitivity (95% CI, 82.82% – 99.82%) and a 78.13% specificity (95% CI, 66.57% – 86.50%) (Supplementary Figs. 1E, F).

These results indicate that the analysis of circulating immune checkpoints and cytokines represents a highly efficient tool to identify not only NENs in general, but also those developed in a particular organ, such as the pituitary gland.

### Identification of pheochromocytomas and paragangliomas based on soluble immunological factors

**Fig. 5:**
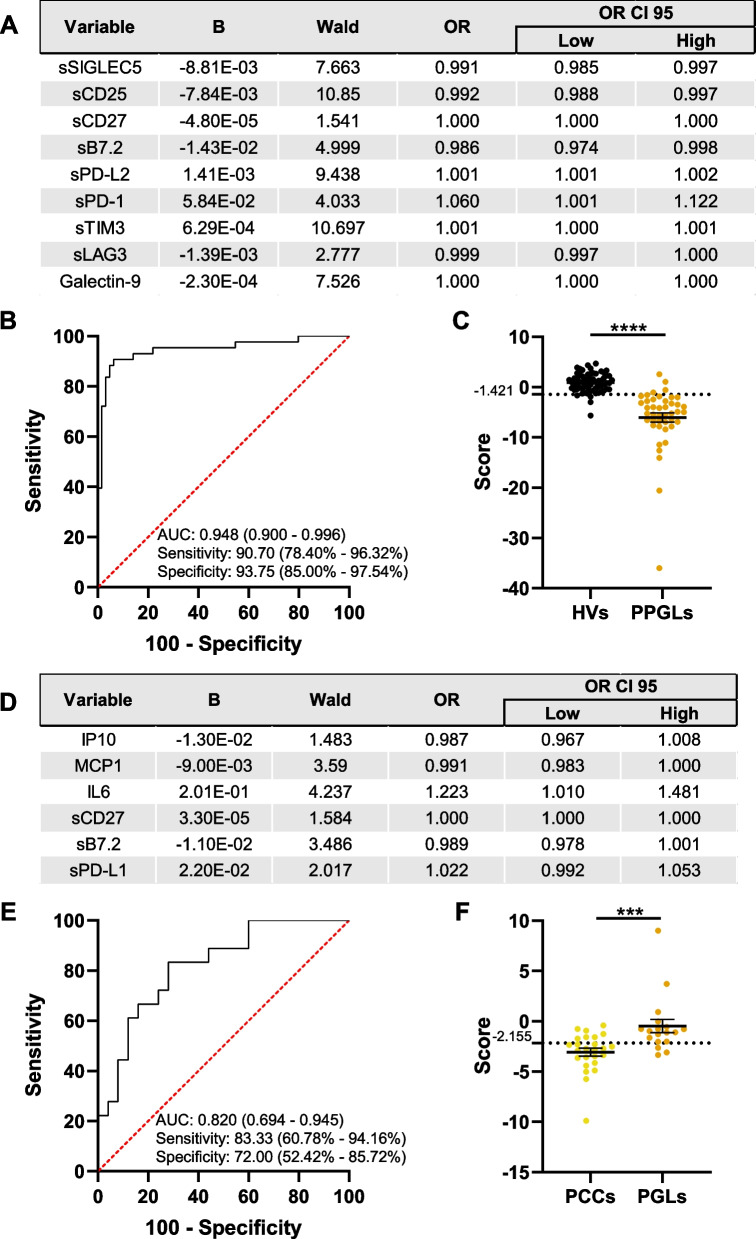
Regression models of circulating immunological factors identify pheochromocytomas and paragangliomas. Wald backward stepwise regressions were performed, including IL4, IL6, IP10, MCP1, sPD-L1, sPD-L2, sPD-1, sCD25, sTIM3, sLAG3, Galectin-9, sCD27, sB7.2 and sSIGLEC5 as variables. **A** Optimal model differentiating neuroendocrine pheochromocytomas and paragangliomas (PPGLs) from healthy volunteers (HVs). **B** ROC curve analysis for identification of PPGLs. **C** Distribution of HVs and patients diagnosed with PPGLs according to the score generated as the optimal Youden index from the ROC curve in **B**. **D** Optimal model differentiating pheochromocytomas (PCCs) and paragangliomas (PGLs). **E** ROC curve analysis for the differentiation between PCCs and PGLs. **F** Distribution of patients suffering from PCCs or PGLs according to the score generated as the optimal Youden index from the ROC curve in **E**. Area under the curves (AUC) are shown in **B** and **E**, as well as sensitivity and specificity. The 95% confidence intervals are shown in brackets. *** *p* < 0.001, **** p < 0.0001, Mann–Whitney test

Afterwards, we wanted to apply the same methodological approach to the identification of PPGLs. The binary regression considered 9 soluble immune checkpoints to build the model (Fig. 5A). Notably, this model was also highly efficient, performing this identification with an AUC of 0.948 (95% CI, 0.900 – 0.996), 90.7% sensitivity (95% CI, 78.40% – 96.32%) and 93.75% specificity (95% CI, 85.00% – 97.54%) (Fig. 5B, C). Next, following the same rationale as before, we run the model considering just immunological factors with a statistically significant difference between HVs and PPGLs in Fig. 1 (IL6, MCP1, sPD-L1, sCD25, sLAG3, Galectin-9 and sB7.2). In this case, the binary regression model included only the five immune checkpoints (Supplementary Fig. 2A). This model was less efficient than the former one, but still provided an acceptable performance with an AUC of 0.856 (95% CI, 0.776 – 0.935), and a sensitivity and specificity of 72.09 (95% CI, 57.31% – 83.25%) and 90.63 (95% CI, 81.02% – 95.63%), respectively (Supplementary Figs. 2B, C).

Remarkably, the binary regression models differentiated HVs from PPGLs, despite PPGLs representing a combined category comprising pheochromocytomas (PCCs) and paragangliomas (PGLs). Therefore, we wanted to evaluate whether these models could distinguish between these two entities. Six circulating immunological factors were included by the regression model to differentiate PCCs and PGLs (Fig. 5D) with an AUC of 0.820 (95% CI, 0.694 – 0.9451), 83.33% sensitivity (95% CI, 60.78% – 94.16%) and 72.00% specificity (95% CI, 52.42% – 85.72%). This performance was weaker than the rest of the models previously analyzed, suggesting a closer relationship between PCCs and PGLs at the circulating immunological profile level.

### Soluble immune checkpoints and cytokines identify gastroenteropancreatic neoplasms and their clinical evolution

**Fig. 6:**
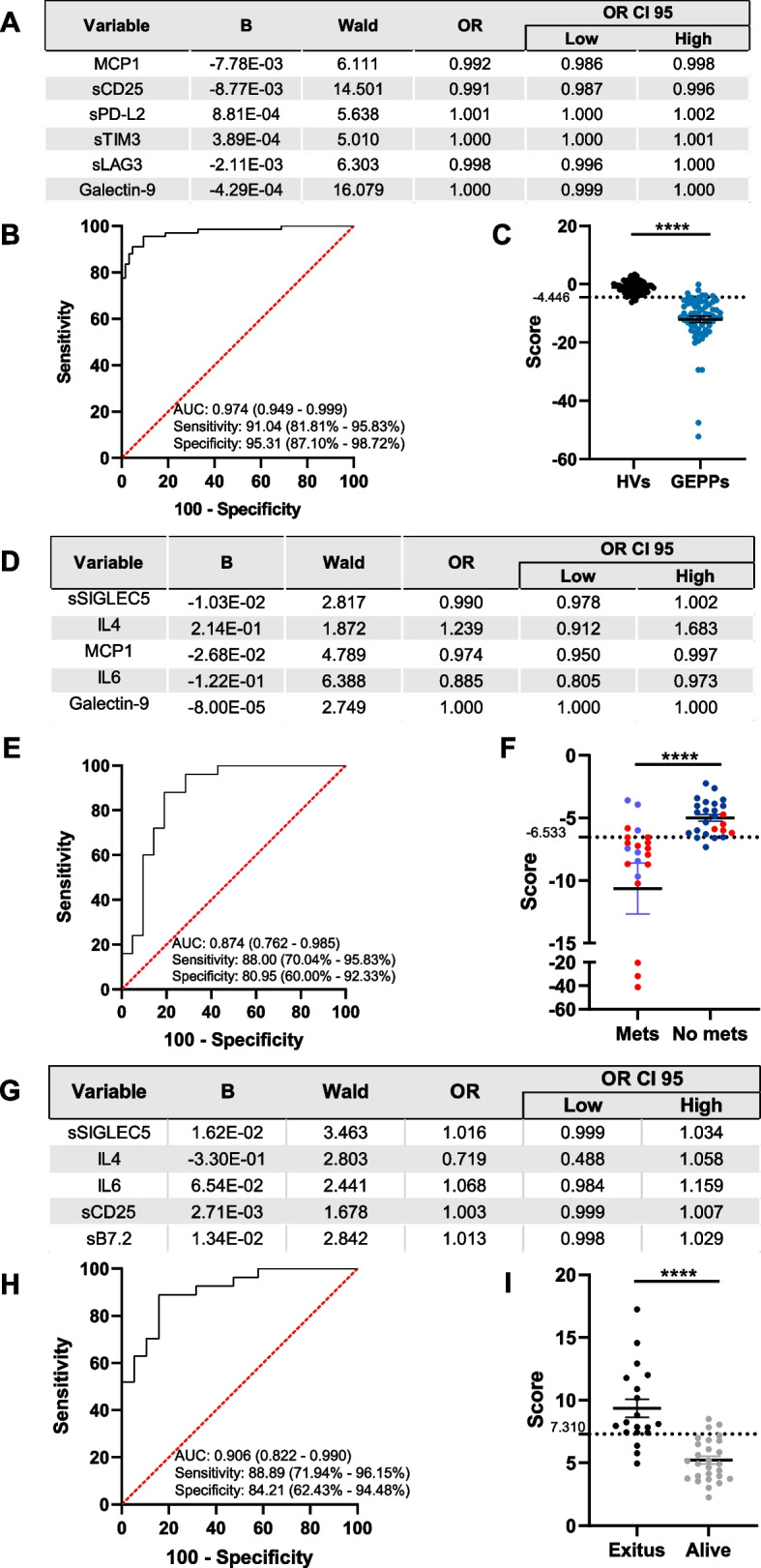
Regression models of circulating immunological factors identify gastroenteropancreatic and pulmonary neuroendocrine neoplasms and their evolution. Wald backward stepwise regressions were performed, including IL4, IL6, IP10, MCP1, sPD-L1, sPD-L2, sPD-1, sCD25, sTIM3, sLAG3, Galectin-9, sCD27, sB7.2 and sSIGLEC5 as variables. **A** Optimal model differentiating gastrointestinal and pulmonary (GEPPs) NENs from healthy volunteers (HVs). **B** ROC curve analysis for identification of GEPPs. **C** Distribution of HVs and patients diagnosed with GEPPs according to the score generated as the optimal Youden index from the ROC curve in **B**. **D** Optimal model differentiating metastatic (Mets) and no metastatic (No mets) GEPP NENs. **E** ROC curve analysis for the differentiation between Mets and No mets GEPP NENs. **F** Distribution of patients suffering from Mets and No mets GEPP NENs according to the score generated as the optimal Youden index from the ROC curve in **E**. Red dots indicate patients with an *exitus* outcome. **G** Optimal model differentiating GEPP NENs according to their outcome (*exitus* or alive). **H** ROC curve analysis for the differentiation between patients suffering from GEPP NENs according to their outcome. **I** Distribution of patients evolved to *exitus* or alive according to the score generated as the optimal Youden index from the ROC curve in **H**. Area under the curves (AUC) are shown in **B**, **E**, and **H**, as well as sensitivity and specificity. The 95% confidence intervals are shown in brackets. **C**, **F**, **** p < 0.0001, Mann–Whitney test. **I** unpaired T test **** p < 0.0001

Next, we evaluated the efficacy of our liquid biopsy approach to the identification of GEPPs NENs. The regression model was highly efficient distinguishing HVs from GEPP NENs based on six circulating immunological factors (Fig. 6A), offering and AUC of 0.974 (95% CI, 0.949 – 0.999) with a 91.04% sensitivity (95% CI, 81.81% – 95.83%) and a specificity of 95.31% (95% CI, 87.10% – 98.72%) (Fig. 6B, C). When this analysis was performed using only those statistically different parameters between HVs and GEPP NENs (Fig. 1), namely, MCP1, sCD25, sLAG3, Galectin-9, sCD27 and sB7.2, the binary regression model included only five immunological factors (Supplementary Fig. 2D). This model also showed a good performance, with an AUC to differentiate HVs from GEPP NENs of 0.959 (95% CI, 0.931 – 0.987), 95.52% sensitivity (95% CI, 87.64% – 98.78%) and 81.25% specificity (95% CI, 70.03% – 88.94%) (Supplementary Fig. 2E, F).

GEPP NENs comprise gastrointestinal, pancreatic and pulmonary neoplasms. We wanted to evaluate whether the levels of circulating immune checkpoints and cytokines could subclassify them. Remarkably, both binary regression models comparing gastrointestinal versus pulmonary NENs showed a 100% sensitivity, 95% CI, 85.13% – 100.00% for gastrointestinal and 95% CI, 90.36% – 100.00% for pancreatic (Supplementary Fig. 3A—F). Nevertheless, this sensitivity was reduced to 68.18% (95% CI, 47.32% – 83.64%) when comparing pancreatic and gastrointestinal NENs (Supplementary Fig. 3G—I). Despite the low number of available pulmonary NENs (*n* = 9) could affect this analysis, these results suggest that the location of the GEPP NENs impacts on the circulating immunological profile, differentiating those developed in the gastrointestinal tract from those in the lung.

In this cohort of GEPP NENs, we had access to clinical data regarding the occurrence of metastasis and survival. Therefore, we assessed whether the pattern of circulating immune checkpoints and cytokines could identify these clinically relevant outcomes. A binary regression model including five immunological factors (Fig. 6D) identified metastatic neoplasms with an AUC of 0.874 (95% CI, 0.762 – 0.985), 88.00% sensitivity (95% CI, 70.04% – 95.83%) and 80.95% specificity (95% CI, 60.00% – 92.33%) (Fig. 6E, F). Notably, this analysis showed a good correlation with the *exitus* outcome of the patients (red dots in Fig. 6F). Indeed, when statistically evaluating survival, a model also comprising five immunological parameters (Fig. 6G) performed even better than for metastasis detection, classifying surviving from *exitus* patients with an AUC of 0.906 (95% CI, 0.822 – 0.990), 88.89% sensitivity (95% CI, 71.94% – 96.15%) and 84.21% specificity (95% CI, 62.43% – 94.48%) (Fig. 6H, I).

Collectively, these data indicate that the patterns of circulating immune checkpoints and cytokines show a powerful capability to identify GEPP neoplasms and predict their clinical progression towards metastatic disease or patient mortality.

### The efficiency of the immunological signatures to identify neuroendocrine neoplasms is sex independent and correlates with clinical data

Having established that immunological signatures built with circulating immune checkpoints and cytokines are highly efficient in identifying NENs, we addressed whether this efficiency was affected by the sex of the analyzed individuals. Noteworthy, the classification capacity of the score differentiating between HVs and the whole cohort of NENs was not affected when these individuals were split by sex (Supplementary Fig. 4A). This was also the case when performing this sex-dependency analysis for Pit-NENs (Supplementary Fig. 4B), PPGLs (Supplementary Fig. 4C) and GEPPs neoplasms (Supplementary Fig. 4D).

We then addressed the relationship between the obtained scores and clinical data from patients suffering NENs. Noteworthy, inverse correlations were found between scores and circulating CgA levels assessed by ELISA. These correlations were statistically significant except for Pit-NENs, which nonetheless, showed the same trend (Supplementary Fig. 5A – 5D). Furthermore, although Pit-NENs and PPGLs were almost all well-differentiated neoplasms, a diversity of grades and stages was identified for GEPPs (see Table 1). The study of correlations between the GEPPs scores and these clinical data also showed a negative association between the score value and the grade and stage of the disease (Supplementary Fig. 5E, F). Therefore, these results indicate that the obtained scores are sensitive to clinically relevant data such as CgA levels and the extent of the pathology, at least for GEPPs.

In summary, the cut-offs generated by the binary regression models are highly efficient in identifying NENs, and their performance does not rely on the sex of studied individuals, while they correlate with clinical data. This is relevant for the application of these immunological signatures as a potential tool in a liquid biopsy approach.

### A minimal common immunological signature identifies different neuroendocrine neoplasms with high efficiency

Aiming to get closer to a clinical application for our results, we considered the potential limitation of using up to fourteen immunological markers for the identification of NENs. Therefore, based on the regression models obtained for the detection of NENs (Fig. 4A), Pit-NENs (Fig. 4D), PPGLs (Fig. 5A) and GEPP NENs (Fig. 6A), we looked for common variables represented in all of them. This resulted in the identification of a minimal immunological signature composed by sCD25, sPD-L2, sTIM3, sLAG3 and Galectin-9, whose performance was evaluated for the identification of all the NENs included in the study.

**Fig. 7:**
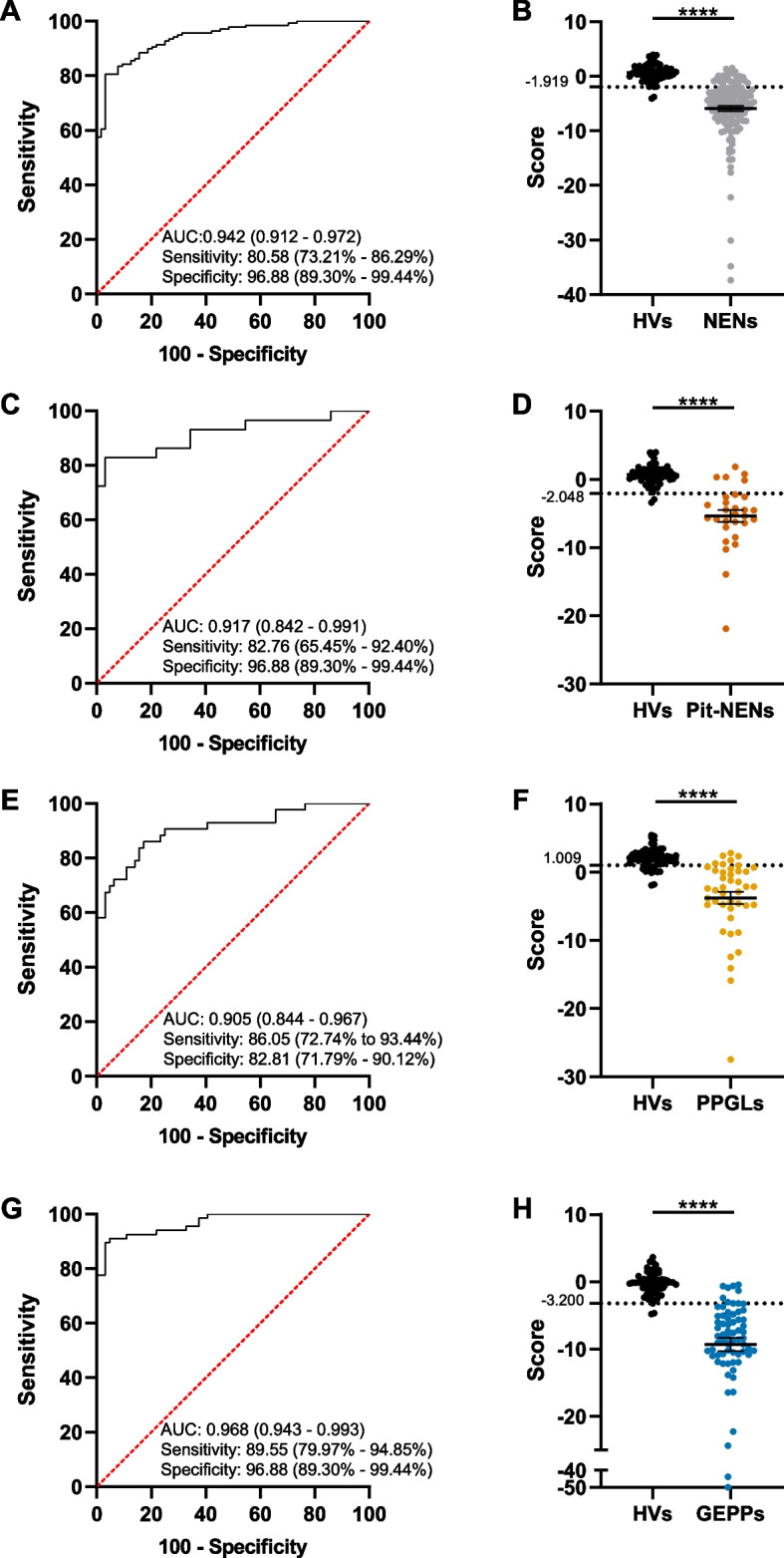
Regression models of circulating immunological factors shared among previous models identify different neuroendocrine neoplasms. Wald backward stepwise regressions, including as variables sCD25, sPD-L2, sTIM3, sLAG3 and Galectin-9, were performed. **A** ROC curve analysis for the differentiation between healthy volunteers (HVs) and patients suffering from neuroendocrine neoplasms (NENs). **B** Distribution of HVs and patients diagnosed with NENs according to the score generated as the optimal Youden index from the ROC curve in **A**. **C** ROC curve analysis for the differentiation between HVs and patients suffering from pituitary NENs (Pit-NENs). **D** Distribution of HVs and patients diagnosed with Pit-NENs according to the score generated as the optimal Youden index from the ROC curve in **C**. **E** ROC curve analysis for the differentiation between HVs and patients suffering from pheochromocytomas and paragangliomas (PPGLs). **F** Distribution of HVs and patients diagnosed with PPGLs according to the score generated as the optimal Youden index from the ROC curve in **E**. **G** ROC curve analysis for the differentiation between HVs and patients suffering from gastroenteropancreatic and pulmonary (GEPPs) NENs. **H** Distribution of HVs and patients diagnosed with GEPPs according to the score generated as the optimal Youden index from the ROC curve in **G**. In **A**, **C**, **E**, and **G**, the area under the curve (AUC), sensitivity and specificity are reported, with 95% confidence intervals shown in brackets. **** *p* < 0.0001, Mann–Whitney test

First, the binary regression model restricted to this signature differentiated HVs from the whole cohort of NENs (Supplementary Fig. 6A) based on a ROC curve with an AUC of 0.942 (95% CI, 0.913 – 0.972), offering a sensitivity of 80.58% (95% CI, 73.21% – 86.29%) and 96.88% specificity (95% CI, 89.30% – 99.44%) (Fig. 7A, B). Therefore, the minimal signature appeared to be highly effective in the identification of NENs.

We next run the regression model to evaluate the identification of Pit-NENs, including only the variables contained in the signature. This analysis (Supplementary Fig. 6B) generated a ROC curve with an AUC of 0.917 (95% CI, 0.842 – 0.991), 82.76% sensitivity (95% CI, 62.45% – 92.40%) and 96.88% specificity (95% CI, 89.30% – 99.44%) (Fig. 7C, D). Applying the same rationale to PPGLs, the minimal signature generated a model (Supplementary Fig. 6C) differentiating these neoplasms from HVs with an AUC of 0.905 (95% CI, 0.844 – 0.967), 86.05% sensitivity (95% CI, 72.74% – 93.44%) and 82.81% specificity (95% CI, 71.79% – 90.12%) (Fig. 7E, F). Eventually, GEPP NENs were efficiently identified based on the minimal signature, with the binary regression model (Supplementary Fig. 6D) providing a ROC curve with an AUC of 0.968 (95% CI, 0.943 – 0.993), 89.55% sensitivity (95% CI, 79.97% – 94.85%) and 96.88% specificity (95% CI, 89.30% – 99.44%) (Fig. 7G, H). These results indicate that the use of a reduced immunological signature based on the quantification of just five soluble immune checkpoints displays a high performance for the identification of different NENs.

**Fig. 8:**
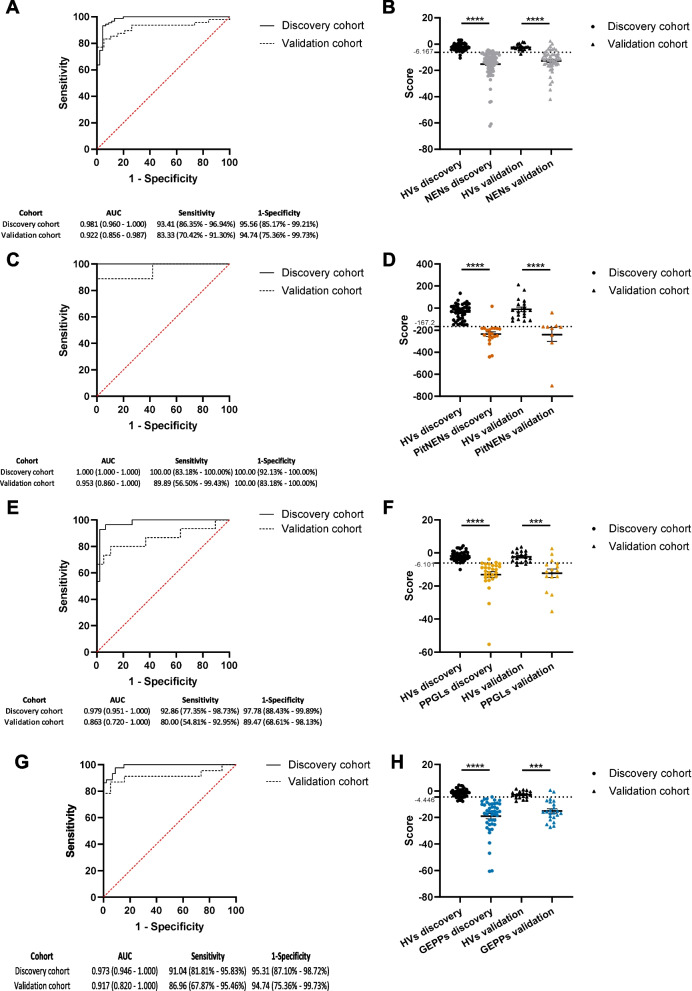
Validation of the minimal immunological signature by a discovery/validation randomization strategy. As indicated in the methods section, samples analyzed in Fig. 7 were randomly split at a 70:30 ratio in discovery and validation cohorts. Wald backward stepwise regressions were performed using samples from the discovery cohorts, including sCD25, sPD-L2, sTIM3, sLAG3, and Galectin-9 as variables. The resulting models were run on samples assigned to the validation cohorts in an unsupervised manner. **A**, **C**, **E**, and **G**: Comparisons of ROC curves built with either the discovery or validation cohorts, for the differentiation between healthy volunteers (HVs) and patients suffering from neuroendocrine neoplasms (NENs) (**A**), pituitary NENs (Pit-NENs) (**C**), pheochromocytomas and paragangliomas (PPGLs) (**E**), and gastroenteropancreatic and pulmonary (GEPPs) NENs (**G**). **B**, **D**, **F**, and **H**: Distribution of HVs and patients diagnosed with NENs (**B**), pituitary NENs (Pit-NENs) (**D**), pheochromocytomas and paragangliomas (PPGLs) (**F**), and gastroenteropancreatic and pulmonary (GEPPs) NENs (**H**) according to the scores generated as the optimal Youden index from each of their respective ROC curves. **A**, **C**, **E**, **G** Area under the curve (AUC) is shown for each of the cohorts, as well as sensitivity and specificity. 95% confidence intervals are shown in brackets. *** *p* < 0.001, **** p < 0.0001, Mann–Whitney test

We then wanted to validate the performance of this minimal immunological signature. Considering the difficulty of collecting new patients suffering from NENs due to their low incidence, we conducted a validation strategy using a random 70/30 split of the samples to generate discovery and validation cohorts, respectively. Thus, samples were randomly divided into discovery and validation sets at a 70:30 ratio. Of note, the internal proportions existing inside each subcohort were meticulously maintained as indicated in the methods section. New regression models, run including only samples randomized as discovery cohorts, generated ROC curves comparable to those using the full cohorts (Supplementary Fig. 7), suggesting the robustness of this approach. Next, the unsupervised application of the new models to the validation cohorts confirmed the great performance of this minimal signature in identifying different NENs with high efficiency, no matter the type of neoplasm analyzed (Fig. 8).

Eventually, we wanted to explore the specificity of this minimal immunological signature in distinguishing NENs from other tumor types. To address this issue, we evaluated the efficiency of this signature identifying NENs *versus* two cohorts of non-NENs: non-small cell lung carcinoma (NSCLC) and luminal breast cancer (see Supplementary Table 1). Compared to the great performance of this minimal signature identifying NENs with an AUC of 0.942, 80.58% sensitivity and 96.88% specificity (see Figs. 7A, B), its differentiating capacity dropped till AUC of 0.769 (95% CI, 0.673 – 0.865), 60.00% sensitivity (95% CI, 46.81% – 71.88%) and 83.33% specificity (95% CI, 68.11% – 92.13%) for NSCLC, with an even worse performance in the case of luminal breast cancer (AUC = 0.658, 95% CI, 0.514 – 0.802; 59.26% sensitivity, 95% CI, 40.73% – 75.49%; 80.56% specificity, 95% CI, 64.97% – 90.25%) (Supplementary Fig. 8). These data indicate that the analysis of the selected immunological factors included in the minimal signature shows a differential high specificity for the identification of NENs.

In summary, Table 2 presents the number of variables included in the binary regression models used to differentiate each of the NENs from HVs, and the accuracy of these models. The accuracy is reported for both the model using all fourteen variables (general signature) and for the minimal signature, which is restricted to the five variables shared across the initial models.

**Table 2:** Accuracies of regression models generated

	Number of variables	Accuracy (%)
General signature		
NENs	11	92.61
Pit-NENs	9	95.74
PPGLs	9	92.52
GEPPs	6	93.12
Minimal signature		
NENs	5	85.71
Pit-NENs	5	92.47
PPGLs	5	84.11
GEPPs	5	93.12

*Abbreviations*: *NENs* Neuroendocrine neoplasms, *Pit-NENs* Pituitary neuroendocrine neoplasms, *PPGLs* Pheochromocytomas and paragangliomas, *GEPP* Gastroenteropancreatic and pulmonary

We can conclude that the analysis of circulating immunological factors such as soluble immune checkpoints and cytokines identifies NENs with an accuracy over 92% when using the whole panel of variables, and between ≈ 85% and ≈ 93% when applying the minimal signature built on just five variables. Therefore, our data support the use of a minimal signature based on soluble immunological factors as a potential liquid biopsy-based tool for the clinical management of NENs.

## Discussion

The results obtained in this work illustrate how the occurrence of NENs has a systemic impact at the immunological level by modulating the levels of circulating soluble immune checkpoints and cytokines. In addition to the identification of all NENs, it is noteworthy that NEN subtypes show individual immunological patterns compared with healthy individuals, thus displaying specificity. For instance, Pit-NENs could be defined by their high expression of MCP1, sCD25, sLAG3, Galectin-9 and sB7.2, while GEPP NENs show elevated levels of IL6, MCP1, sPD-L1, sCD25, sLAG3, Galectin-9, sCD27 and sB7.2. Furthermore, this specificity allows the differentiation between NENs, with GEPP NENs showing the highest sCD25 and sCD27 levels, while PPGLs show the most abundant levels of sSIGLEC5. These results have, at least, two implications. On the one hand, the biological information generated could help to understand the pathophysiology of these rare neoplasms, providing clues to potential new therapies. On the other hand, the identification of specific circulating profiles appears to be a powerful diagnostic and even prognostic tool based on a liquid biopsy approach.

It is important to highlight that the tumors studied in this work represent rare neoplastic entities considered as a such by the RARECARE project, with an annual incidence below 6 people per 100,000 citizens in the European Union, showing a 0.5% overall incidence [1]. In detail, by 2017, the incidence for the NENs studied in this work was 0.04% for Pit-NENs, 0.06% for PPGLs and 1.71% for combined GEPP NENs [1]. Along with their scarcity, the diagnosis of these tumors is really challenging as their symptoms are usually not specific and the clinical manifestations outstand only when metastatic disease is already present [6]. Therefore, new tools for NENs early diagnosis are required, and their impact at the immunological level could provide them.

Along these lines, the immunological landscape in PPGLs and GEPP NENs has begun to gain attention when analyzing tumor biopsies in high-throughput transcriptomic studies [22, 29, 30]. These studies have been seminal to highlight the relevance of the tumor immune infiltration for the evolution of these neoplasms. Indeed, local expression of PD-1/PD-L1 within tumors arises as a potential biomarker of NENs evolution [31, 32], even providing mechanistic insights related to the immunosuppressive phenotype elicited by these neoplasms [26].

Nevertheless, the analysis of circulating immunological factors has been barely tested in the context of GEPP NENs, evaluating cytokines or immune-related growth factors such as the dendritic cell-promoting FLT3L [33, 34]. Our results corroborate to some extent these findings, showing increased levels of cytokines such as IL6 [33], further extending the analysis to circulating soluble immune checkpoints. Of note, the evaluation of these soluble immune checkpoints has been revealed as a powerful tool for cancer diagnosis and even to predict response to therapies [35]. Furthermore, considering the strength of multiparametric analysis as diagnostic tool in different contexts [36, 37], we applied binary regression models to maximize the categorization capacity of individual parameters, as performed before to identify different pathological conditions such as cancer relapse and diagnosis, or infectious severity [16, 28, 33].

Interestingly, despite the high efficiency of these regression models to identify PPGLs, PCCs and PGLs showed comparable patterns between each other. Indeed, the model displayed one of the poorest performances all through the study, suggesting that both NENs represent two similar tumor entities. In fact, these two neoplasms share embryonic origin, and both derive from chromaffin cells, with a key difference, as PCCs are confined to the adrenal medulla, while PGLs are located in extra-adrenal paraganglia [29]. Therefore, location along the body shows a minimal impact on the systemic immunological behavior of these pathophysiologically-related neoplasms.

Nonetheless, the circulating immunological profile appears to classify pancreatic and gastrointestinal NENs as digestive tract neuroendocrine neoplasms, while differentiating them from pulmonary. Hence, the circulating pattern of soluble immune checkpoints and cytokines identifies these tumor locations as differential entities, supporting their specific clinical management. Indeed, the last classification of neuroendocrine neoplasms by the WHO in 2022 established different classification criteria for NENs of the gastrointestinal and pancreatobiliary tract than for those located at the lungs and thymus [7]. Consequently, in this case, the circulating immunological profile provides relevant information regarding the location of the neoplasm.

In this sense, considering that levels of soluble immune checkpoints are being evaluated as indicators of clinical response to immune checkpoint inhibitors (ICIs), and that immunotherapy based on these agents is being assayed against NENs in multiple clinical trials [4, 38, 39], it would be interesting to study the evolution of these circulating immunological factors as potential biomarkers for patients’ response or selection.

Highlighting some of the immunological parameters analyzed in this study, sCD25 emerges as one of the most consistently included factor in the diagnostic models, with statistical power in terms of odds ratio. sCD25 represents the soluble form of the IL2 receptor. It is generated through the shedding of the cell surface receptor, and although different proteases have been involved, the actual mechanism is not fully understood [40]. Most of the available information indicates an immunosuppressive function for sCD25, favoring the development of T regulatory cells [41]. From the clinical point of view, increased sCD25 levels have been found in different pathologies, such as sepsis [42] or the hyperinflammatory syndrome hemophagocytic lymphohistiocytosis [43], mostly related to poor outcomes [44]. Our results indicate that sCD25 levels are increased in all the analyzed NENs, supporting a potential “cold” immunosuppressive phenotype for these neoplasms [26, 29, 45]. Interestingly, sCD25 levels were even higher in GEPP NENs. Considering the tight relationship between T regulatory cells and the tolerance maintenance at mucosae [46], these data warrant future investigations to better understand of the pathophysiology of GEPP NENs.

sSIGLEC5 is another relevant soluble immunological factor to consider, included in the prognostic models for GEPP NENs. SIGLEC5 is an inhibitory immune checkpoint expressed both in myeloid cells such as monocytes and neutrophils, as well as in T cells [47]. sSIGLEC5 is generated by proteolytic cleavage from the cell surface [48]. Of note, increased sSIGLEC5 circulating levels have been widely described as a bad prognosis factors in different pathologies such as sepsis [48], myocarditis [49], colorectal cancer [50] or lung cancer relapse [16]. Therefore, SIGLEC5 might not only have a potential prognostic value in neuroendocrine neoplasms, but it could also be implicated in the mechanisms leading to malignant transformation and the resulting poorer clinical outcomes in these tumors.

Beyond the potential biological information inferred from our results, the most relevant application generated in this study is a liquid biopsy-based tool to identify NENs. Current diagnosis of these neoplasms relies on tissue biopsies and biochemical analyses, that can be general or specific for certain NENs such as gastrin for gastrinomas, coupled to imaging techniques [8].

Among the common soluble biomarkers, determination of CgA remains the gold-standard. However, these markers are elevated in only about 50% of patients with NENs [11, 13]. In detail, regarding CgA, different studies have reported sensitivities ranging from 32 to 92%, depending on the type of NEN, secretory condition and tumor burden [51]. A meta-analysis including 13 heterogeneous studies using CgA as a diagnostic marker for NENs versus healthy controls reported a 73% sensitivity with 95% specificity [53]. A more recent study comparing various commercially available ELISA kits for CgA determination declared sensitivities between 41.2% and 64.7%, with specificity ranging from 69.6% to 82.6% [53]. Overall, these data instigate the need for more precise blood markers. In this context, the use of hematological parameters as diagnostic or prognostic tools for NENs has been reported. Early studies showed increased leukocyte counts as inflammation marker in PCCs compared to other forms of hypertension [21]. These findings promoted the use of the neutrophil-to-lymphocyte ratio (NLR) as a predictive factor of clinical outcomes in pancreatic NENs, although it showed poor sensitivity and specificity [18, 19].

One of the most promising diagnostic strategies nowadays using blood for the identification of NENs is the so-called NET-specific gene transcript analysis (NETest). This is a blood-based multiparametric molecular assay based on the detection of 51 markers by polymerase chain reaction (PCR) [53]. The performance of this test is remarkable. In one study NETest detected PPGLs with a 100% sensitivity and 92% specificity [53]. Another study determined a 96% concordance between positive diagnosis by NETest and demonstrable disease by imaging, outperforming CgA in the identification of GEPP NENs [53], with a 98% sensitivity and 66% specificity in an alternative study [53]. This performance is comparable to the results obtained in our study. Nonetheless, this method shows a relevant limitation. NETest is a molecular biology-based technique that requires a specific 3-step protocol before the test is run (RNA isolation, cDNA production and PCR) [53]. This tedious procedure motivated the investigation of ready-to-use PCR systems [53]. Overcoming this issue, our approach can be easily performed, directly in blood samples, using cellular biology-based methodologies such as ELISA. Furthermore, our results provide also noteworthy accuracy of around 93% when considering the whole panel of immunological markers, and of approximately 90% if operating with the five-parameters minimal signature. Considering all these issues, we have tested the simplest signature based on only five parameters, representing a potentially easily implementable diagnostic approach, with a remarkable performance in terms of diagnostic accuracy. Of note, despite an internal validation has been performed based on a sample randomization strategy and unsupervised application of this minimum signature, the clinical validity of this approach and its specificity in NENs *versus* non-NENs tumors requires further validation in independent cohorts.

## Conclusions

Our findings demonstrate that the presence of NENs significantly alters the systemic soluble immunological profile of patients. Exploiting this concept, we propose the analysis of soluble immune checkpoints and cytokines in a blood liquid biopsy for the identification of NENs. Besides, this test may offer prognostic applications. This approach could be easily evaluated and, eventually, implemented in the diagnostic routine for NENs, complementing imaging techniques to enhance the identification and management of these complex neoplasms.

## Supplementary Information

Additional file 1.

## Data Availability

The dataset used and analyzed during the current study are available from the lead contact author on reasonable request.

## Abbreviations

AUC: Area under the curve
CgA: Chromogranin-A
ELISA: Enzyme-linked immunoassay
GEPP: Gastroenteropancreatic
ICIs: Immune Checkpoint inhibitors
NENs: Neuroendocrine neoplasms
NsE: Neuron-specific enolase
PCCs: Pheochromocytomas
PCR: Polymerase chain reaction
PGLs: Paragangliomas
PPGLs: Pheochromocytomas and paragangliomas
WHO: World Health Organization
