# Supplementary material for: A blood-based liquid biopsy analyzing soluble immune checkpoints and cytokines identifies distinct neuroendocrine tumors
Source: J Exp Clin Cancer Res. 2025 Mar 5;44:82. doi: 10.1186/s13046-025-03337-3 (PMC11881345; doi:10.1186/s13046-025-03337-3)
Supplement: Supplementary file 1 — Additional file 1. [file 13046_2025_3337_MOESM1_ESM.docx]

**Supplementary Figure 1.** **Regression models of statistically different circulating immunological factors identify NENs.**

**A** Wald backward stepwise regression was performed, including IL6, MCP1, sPD-L1, sPD-L1, sCD25, sLAG3, Galectin-9, sCD27 and sB7.2 as variables. The optimal model differentiating neuroendocrine neoplasms (NENs) from healthy volunteers (HVs) is shown. **B** ROC curve analysis for identification of NENs. **C** Distribution of HVs and patients diagnosed with NENs according to the score generated as the optimal Youden index from the ROC curve in B. **D** Wald backward stepwise regression was performed, including MCP1, sCD25, sLAG3, Galectin-9 and sB7.2 as variables. The optimal model differentiating pituitary NENs (Pit-NENs) from healthy volunteers (HVs) is shown. **E** ROC curve analysis for identification of Pit-NENs. **F** Distribution of HVs and patients diagnosed with Pit-NENs according to the score generated as the optimal Youden index from the ROC curve in E. B, E, Area under the curve (AUC) is shown, as well as sensitivity and specificity. 95% confidence intervals are shown in brackets. **** *p* < 0.0001, Mann-Whitney test.

**Supplementary Figure 2.** **Regression models of statistically different circulating immunological factors identify PPGLs and GEPP NENs.**

**A** Wald backward stepwise regression was performed, including IL6, MCP1, sPD-L1, sCD25, sLAG3, Galectin-9 and sB7.2 as variables. The optimal model differentiating neuroendocrine pheochromocytomas and paragangliomas (PPGLs) from healthy volunteers (HVs) is shown. **B** ROC curve analysis for identification of PPGLs. **C** Distribution of HVs and patients diagnosed with PPGLs according to the score generated as the optimal Youden index from the ROC curve in B. **D** Wald backward stepwise regressions was performed, including MCP1, sCD25, sLAG3, Galectin-9, sCD27 and sB7.2 as variables. The optimal model differentiating gastroenteric and pulmonary (GEPPs) NENs from healthy volunteers (HVs) is shown. **E** ROC curve analysis for identification of GEPPs. **F** Distribution of HVs and patients diagnosed with GEPPs according to the score generated as the optimal Youden index from the ROC curve in E. **** *p* < 0.0001, Mann-Whitney test.

**Supplementary Figure 3.** **Regression models of circulating immunological factors between different locations of GEPP NENs.**

Wald backward stepwise regressions were performed, including IL4, IL6, IP10, MCP1, sPD-L1, sPD-L2, sPD-1, sCD25, sTIM3, sLAG3, Galectin-9, sCD27, sB7.2 and sSIGLEC5 as variables. **A** Optimal model differentiating pulmonary (Pulm) from gastrointestinal (GasInt) NENs. **B** ROC curve analysis for identification of Pulm and GasEnt NENs. **C** Distribution of Pulm and GasInt NENs according to the score generated as the optimal Youden index from the ROC curve in B. **D** Optimal model differentiating pulmonary (Pulm) from pancreatic (Panc) NENs. **E** ROC curve analysis for identification of Pulm and Panc NENs. **F** Distribution of Pulm and Panc NENs according to the score generated as the optimal Youden index from the ROC curve in E. **G** Optimal model differentiating pancreatic (Panc) from gastrointestinal (GasInt) NENs. **H** ROC curve analysis for identification of Panc and GasInt NENs. **I** Distribution of Panc and GasInt NENs according to the score generated as the optimal Youden index from the ROC curve in H. C, F, ** *p* < 0.01, **** *p* < 0.0001, Unpaired T test, I, *** *p* < 0.001, Mann-Whitney test.

**Supplementary Figure 4.** **The efficiency of the immunological signatures to identify neuroendocrine neoplasms is sex independent.**

Regression models presented in Figures 4A, 4D, 5A and 6A were applied to the whole cohort of neuroendocrine neoplasms (NENs), pituitary NENs (Pit-NENs), pheochromocytomas and paragangliomas (PPGLs) and gastroenteropancreatic and pulmonary (GEPPs) NENs, differentiating between men (♂) and women (♀). **** *p* < 0.0001, Mann-Whitney test.

**Supplementary Figure 5. Correlation between obtained scores and clinical characteristics.** Correlations were established between the scores presented in Figures 4C (for patients with neuroendocrine neoplasms (NENs) (**A**), 4F (for patients with pituitary (Pit-NENs) (**B**), 5C (for patients with pheochromocytomas and paragangliomas (PPGLs) (**C**), and 6C (for patients with gastroenteropancreatic and pulmonary (GEPPs) NENs (**D**) and circulating chromogranin A (CgA) levels. Correlations were established between the scores generated in Figure 6C for patients suffering from GEPPs and the grade (**E**) and stage (**F**) of the disease. Spearman r coefficient and *p* values are shown for each correlation. * *p* < 0.05, ** *p* < 0.01, **** *p* < 0.0001, **** *p* < 0.0001.

**Supplementary Figure 6.** **Regression models of circulating immunological factors shared among previous models.**

Wald backward stepwise regressions were performed, including sCD25, sPD-L2, sTIM3, sLAG3 and Galectin-9 as variables. The optimal model differentiating **A** the whole cohort of neuroendocrine neoplasms (NENs), **B** pituitary NENs (Pit-NENs), **C** pheochromocytomas and paragangliomas (PPGLs), and **D** gastroenteric and pulmonary (GEPPs) NENs from healthy volunteers (HVs) are shown.

**Supplementary Figure 7. Samples randomization generates comparable ROC curves between full and discovery cohorts.** As indicated in the methods section, samples analyzed in Figure 7 were randomly divided into discovery and validation cohorts at a 70:30 ratio. Wald backward stepwise regressions, including sCD25, sPD-L2, sTIM3, sLAG3, and Galectin-9 as variables, were performed exclusively on samples assigned to the discovery cohorts. The models obtained were compared with those obtained in Figure 7, which include the full cohorts for each pathology. The comparison of ROC curves for the differentiation between healthy volunteers (HVs) and neuroendocrine neoplasms (NENs) (**A**), pituitary NENs (Pit-NENs) (**B**), pheochromocytomas and paragangliomas (PPGLs) (**C**), and gastroenteric and pulmonary (GEPPs) NENs (**D**) are depicted. Area under the curve (AUC) is shown for each cohort, along with sensitivity and specificity. The 95% confidence intervals are provided in brackets.

**Supplementary Figure 8.** **Comparative performance of the minimal common immunological signature across diverse cancers. A** Wald backward stepwise regressions, including as variables sCD25, sPD-L2, sTIM3, sLAG3 and Galectin-9, were performed. ROC curve analysis for the differentiation between healthy volunteers (HVs) and patients suffering from neuroendocrine neoplasms (NENs) (grey line), non-small cell lung carcinoma (NSCLC) (green line) and luminal breast cancer (LBC) (pink line) are depicted. Area under the curve (AUC) is shown for each of the cohorts, along with sensitivity and specificity. The 95% confidence intervals are shown in brackets. **B** Distribution of patients suffering from NENs, NSCLC, and LBC according to the score generated as the optimal Youden index from the ROC curve in A. * *p* < 0.05, **** *p* < 0.0001, Kruskal-Wallis test.

**Supplementary Table 1.** Demographic and clinical data of NSCLC and luminal breast cancer patients.

| Variable | n | % |
| --- | --- | --- |
| Number of samples | 118 | 100 |
| Healthy volunteers  Non-small cell lung carcinoma (NSCLC) patients | 36  55 | 30.51  46.61 |
| Luminal breast cancer patients | 27 | 22.88 |
| Age, median, years (min-max) |  |  |
| Healthy volunteers  Non-small cell lung carcinoma (NSCLC) patients | 53.5 (30-67)  70.0 (59-84) | -  - |
| Luminal breast cancer patients | 67.0 (53-82) | - |
| Sex |  |  |
| Healthy volunteers  Male  Female  Non-small cell lung carcinoma (NSCLC) patients  Male  Female  Luminal breast cancer patients  Male  Female | 36  31  5  55  34  21  27  2  25 | 30.51  86.11  13.89  46.61  61.82  38.18  22.88  7.41  92.59 |
|  |  |  |
